# Intra-articular injection of synovial mesenchymal stem cells improves cartilage repair in a mouse injury model

**DOI:** 10.1038/srep23076

**Published:** 2016-03-17

**Authors:** J. Mak, C. L. Jablonski, C. A. Leonard, J. F. Dunn, E. Raharjo, J. R. Matyas, J. Biernaskie, R. J. Krawetz

**Affiliations:** 1 McCaig Institute for Bone & Joint Health, University of Calgary, Calgary, AB, Canada; 2 University of Calgary, Department of Surgery, Calgary, AB, Canada; 3 University of Calgary, Department of Radiology, Calgary, AB, Canada; 4 Experimental Imaging Centre, University of Calgary, Calgary, AB, Canada; 5 University of Calgary, Department of Comparative Biology and Experimental Medicine, Calgary, AB, Canada; 6 Alberta Children’s Hospital Research Institute, University of Calgary, Calgary, AB, Canada; 7 University of Calgary, Department of Anatomy and Cell Biology, Calgary, AB, Canada

## Abstract

Controversy remains whether articular cartilage has an endogenous stem/progenitor cell population, since its poor healing capacity after injury can lead to diseases such as osteoarthritis. In the joint environment there are mesenchymal stem/progenitor cells (MSCs) in the synovial membrane and synovial fluid that can differentiate into cartilage, but it is still under debate if these cells contribute to cartilage repair *in vivo*. In this study, we isolated a Sca-1 positive, chondrogenesis capable population of mouse synovial MSCs from C57BL6 and MRL/MpJ “super-healer” strains. Intra-articular injection of Sca-1 + GFP + synovial cells from C57BL6 or MRL/MpJ into C57BL6 mice following cartilage injury led to increased cartilage repair by 4 weeks after injury. GFP expression was detected in the injury site at 2 weeks, but not 4 weeks after injury. These results suggest that synovial stem/progenitor cells, regardless of strain background, have beneficial effects when injected into an injured joint. MSCs derived from MRL/MpJ mice did not promote an increased repair capacity compared to MSCs derived from non-healing C57BL6 controls; however, MRL/MpJ MSCs were observed within the defect area at the time points examined, while C57BL6 MSCs were not.

Osteoarthritis (OA) is characterized by the progressive loss of articular cartilage, likely due to the intrinsic inability of cartilage to heal itself satisfactorily. In humans, endogenous cartilage repair is ineffective, and articular defects deep enough to reach the marrow cavity become filled with fibrocartilage. The biomechanical properties of fibrocartilage are insufficient to provide a durable and lasting repair of the articulating surface. However, the recent characterization of stem cells in the joint environment (synovium and synovial fluid) that are able to differentiate into chondrocytes suggests that an intrinsic repair mechanism may exist.

Mesenchymal stem cells (MSCs) have been isolated from a variety of tissues including bone marrow (BM)^1^, adipose tissue^2^, synovium and synovial fluid^3^ and periosteum^4^. Indeed, the use of BM-MSCs for cartilage repair or in cartilage tissue engineering is still prevalent and remains the gold standard^5^,^6^,^7^,^8^,^9^. However, it might be more effective to utilize a progenitor/stem cell that is developmentally more closely related to chondrocytes. During the development of synovial joints in mice and humans, the synovium and cartilage originate from a common pool of cells^10^,^11^. MSC populations have been derived from the synovium and synovial fluid and there is compelling evidence that these cells possess cartilage repair potential^12^,^13^,^14^. Additionally, *in vivo* studies have shown that endogenous cells from synovial membrane can contribute to partial-thickness cartilage defect repair^15^,^16^,^17^. While the cell surface markers that identify MSC populations are still under debate, a number of studies have demonstrated that Stem Cell Antigen 1 (Sca1) and Platelet-derived growth factor receptor - alpha (PDGFRa/CD140a) double positive cells from murine bone marrow^18^,^19^,^20^ or synovium^15^,^21^ have multipotent differentiation capacity.

To advance the field of cartilage regeneration, it is necessary to understand the natural progression of repair before potential therapeutic targets can be identified. The goal of this project is therefore to understand the healing process of cartilage after injury using a model system that demonstrates endogenous cartilage repair. In 2008, it was reported that MRL/MpJ (MRL) mice were able to repair full thickness lesions in the articular cartilage of the trochlear groove^22^. Specifically, lesions in MRL mice displayed ample chondrocytes, proteoglycan, collagen II and collagen VI whereas lesions in C57BL6 mice exhibited lower levels of proteoglycan and collagen II. At 4 and 8 weeks, MRL mice appear to be protected from the morphological changes that occur in the C57BL6 mice in response to traumatic injury. Like patients afflicted with osteoarthritis, reduced bone density and thickening of the subchondral bone was seen only in C57BL6 mice^23^. Furthermore, the authors observed significant cartilage degeneration compared to the uninjured contralateral limb in C57BL6 mice and not in MRL mice. A comparison of full thickness and partial thickness cartilage lesions in C57BL6 and MRL mice has also been undertaken at 6 and 12 weeks^22^ and the authors observed that partial thickness lesions did not heal in either strain. Full thickness lesions, however, resulted in significant repair in MRL mice at both 6 and 12 week time-points. Morphologically, the repair tissue contained chondrocytes and was comprised of proteoglycans and collagen. It is important to note that in both the Ward *et al.*^23^ study and Fitzgerald^22^ study, only morphological changes were observed and no attempt was made to elucidate the repair response at a cellular level: specifically, if stem cells and/or immune cells (such as macrophages) are playing any role in the enhanced regeneration in MRL mice.

The first investigation into which cell types are initially activated in response to articular injury was published in 2011^15^. The authors induced a full-thickness joint surface injury in C57BL6 mice and evaluated the cellular response 4, 8, and 12 days post injury. Using DNA label retention, at 4 and 8 days after injury the authors observed increased proliferation of slow-cycling cells in the synovium that stained negative for hematopoietic markers but positive for MSC markers. At 12 days post injury, the authors observed the presence of double-labelled cells that also expressed chondrogenic markers in the cartilage. Furthermore, the study utilized C57BL6 animals, which have previously been documented to have poor cartilage repair capacity^24^. In another study, the authors investigated whether intra-articular transplantation of bone marrow-derived MSCs from MRL mice were superior in slowing down the post-traumatic arthritis compared to bone marrow-derived MSCs from C57BL6 mice^25^. The authors observed an overall protective effect from stem cell treatment, independent of which mouse strain the MSCs were derived from. The authors also observed a small subset of transplanted cells that had engrafted into the injury site. Since MRL mice have the capacity to repair cartilage, the similarity between MRL and C57BL6 MSC repair capacity in this *in vivo* study seems paradoxical. However, synovial MSCs from MRL mice may demonstrate increased cartilage repair capacity compared to C57BL6 mice, since synovial stem/progenitor cells have increased *in vitro* chondrogenic capacity when compared to bone marrow stem/progenitors^26^,^27^.

In this study we first characterized which cell populations were involved in endogenous cartilage healing in MRL mice and then transplanted these cells into C57BL6 non-healing mice to observe if MRL “superhealer” progenitor cells were capable of promoting increased regenerative capacity of articular cartilage.

## Results

### Characterizing Endogenous Response to Cartilage Injury

**Figure 1:**
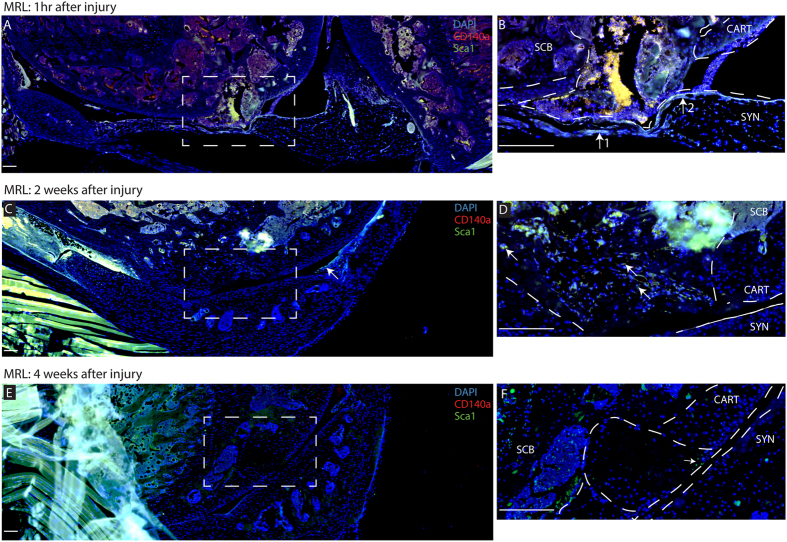
Characterization of Endogenous MRL MSCs after Cartilage Injury. Immediately after joint injury Sca-1^+^CD140a^+^ cells can be observed within the defect and the adjacent synovium (arrow **B**) as well as sub-chondral bone. At 2 weeks after injury, Sca-1^+^ cells can be observed in the defect, but are no longer staining positive for CD140a (arrows **D**). By 4 weeks after injury, very few Sca-1^+^ cells can be found in or around the defect area (arrow **F**). Scale bars = 200 μm. SCB = sub-chondral bone, CART = cartilage, SYN = synovium.

**Figure 2:**
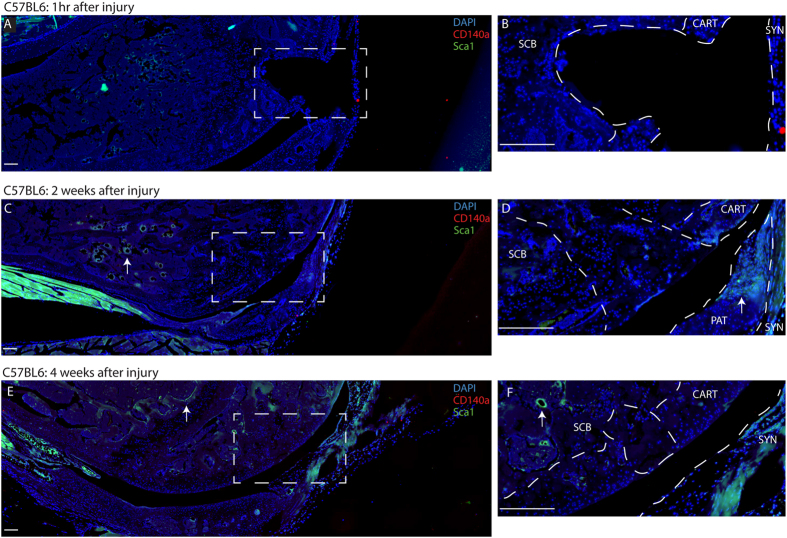
Characterization of Endogenous C57BL6 MSCs after Cartilage Injury. Immediately after joint injury neither Sca-1^+^ nor CD140a^+^ cells can be observed around the defect area or in the adjacent synovium or sub-chondral bone. At 2 weeks after injury Sca-1^+^ cells can be observed in sub-chondral bone, and appear to be associated with the vasculature (arrow **C**). At this time-point Sca-1^+^ cells can also be seen in the patella (arrow **D**). By 4 weeks after injury, no Sca-1^+^ cells can be found in or around the defect area, however, Sca-1^+^ cells can still be observed in the sub-chondral bone, associated with blood vessels (arrow **E**,**F**). Scale bars = 200 μm. SCB = sub-chondral bone, CART = cartilage, SYN = synovium, PAT = patella.

To assess the presence of synovial MSCs after full-thickness cartilage injury in healing (MRL) and non-healing (C57BL6) strains, histology was used to examine the defect immediately after the injury, and 2 or 4 weeks after injury. In MRL mice, Sca-1 + CD140a+ cells were observed in the defect and adjacent synovium immediately after injury (Fig. 1, Supplementary Figure 1); however, a Sca-1 + CD140− population was also observed (Supplementary Figure 1). At later time-points (2 and 4 weeks after injury) Sca-1 + CD140+ were not observed and only Sca-1 + CD140− cells were seen in proximity to the defect (Fig. 1). In C57BL6 mice, only Sca-1+ cells were observed in and around the defect site at the time-points examined (Fig. 2). Additionally, there was more extensive staining in the sub-chondral bone and marrow compartments in MRL mice vs. C57BL6 mice at all time-points examined (Figs 1 and 2). Sca-1 expression was confined to (and in close proximity to) blood vessels in the marrow (Fig. 2). Interestingly, uninjured C57BL6 mice showed limited CD140a expression in their joints, while there was robust Sca-1 and CD140 staining in uninjured MRL knee joints (Supplementary Figure 2).

**Figure 3:**
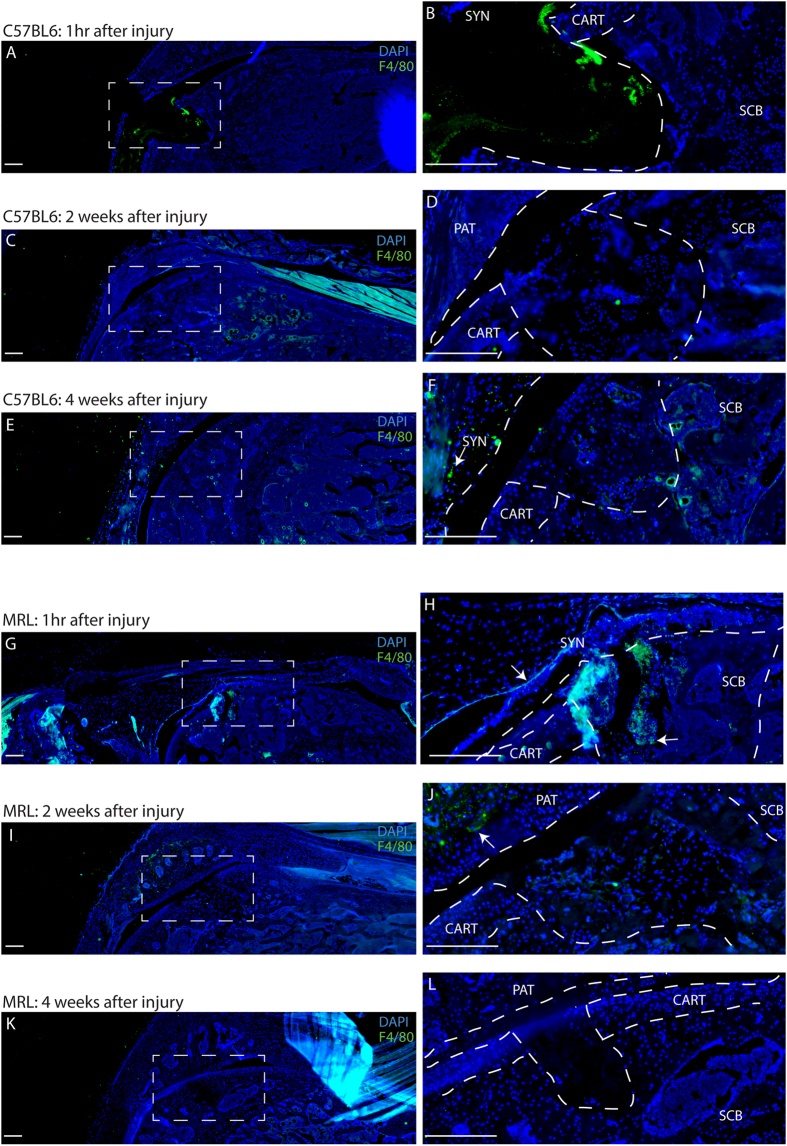
Macrophage identification in C57BL6 and MRL joints after Cartilage Injury. In C57BL6 mice, no F4/80 positive macrophages are observed in the defect area until 4 weeks after cartilage injury (arrow). In MRL mice, F4/80 positive macrophages are observed in the defect and synovium immediately after injury (arrow) and F4/80 positive cells decrease in weeks 2 and 4 after injury. Scale bars = 200 μm. SCB = sub-chondral bone, CART = cartilage, SYN = synovium, PAT = patella.

In addition to MSCs, the localization of macrophages was also examined after injury in both strains. In MRL mice, F4/80 positive cells (a common pan-macrophage marker^28^) could be seen in and around the defect after injury; however, very few could be observed at 2 or 4 weeks after injury (Fig. 3). In C57BL6 mice, no F4/80 positive cells (staining present in the defect appears to lack nuclei) were observed at the defect site until the 4 week time-point after injury (Fig. 3). Opposite to what was observed with MSC markers in uninjured joints, increased F4/80 expression was detected in uninjured C57BL6 knee joints compared to the MRL strain (Fig. 3).

### Characterizing the Potential of MSCs *in Vitro*

**Figure 4:**
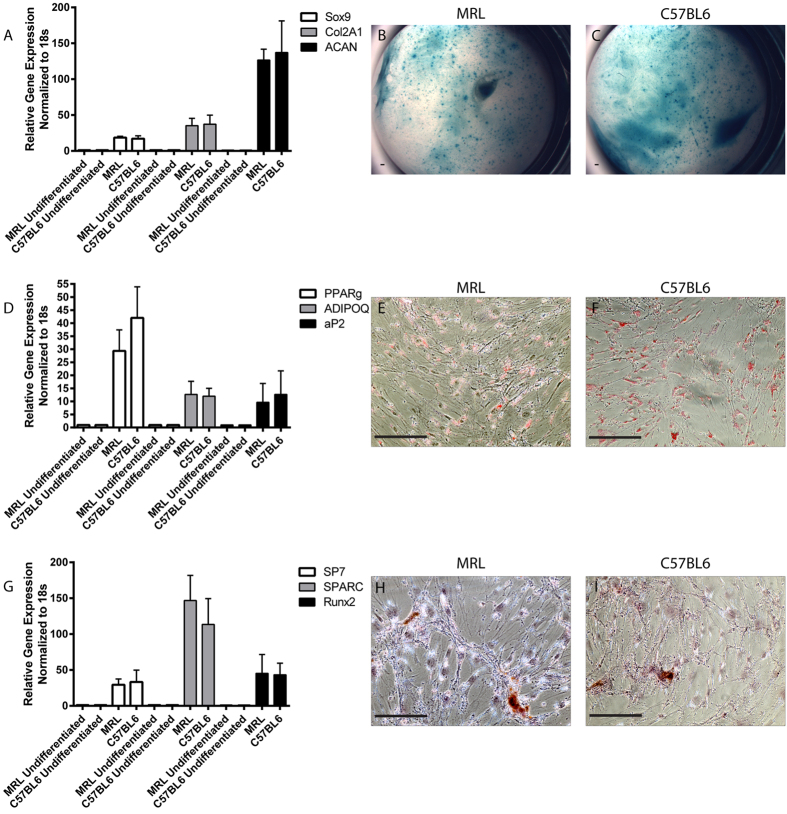
Characterization of MSCs from MRL and C57BL6 mice *In Vitro*. Synovial MSCs were harvested from MRL and C57BL6 mice and examined for chondrogenic (**A–C**), adipogenic (**D–F**) and osteogenic (**G–I**) potential using qRT-PCR for specific markers (**A**,**D**,**G**) and histological stains (**B**,**C**,**E**,**F**,**H**,**I**). No differences in multipotent differentiation capacity was observed between MSCs derived from the two strains. Scale bars = 100 μm.

Since there was a difference in the response of endogenous MSCs from MRL and C57BL6 mice to the cartilage injury *in vivo*, MSCs were harvested from the synovium of both strains and their differentiation potential was compared *in vitro* (Fig. 4). Synovial MSCs from MRL and C57BL6 mice demonstrated a similar capacity to differentiate into chondrocytes (Fig. 4A–C), adipocytes (Fig. 4D–F) or osteoblasts (Fig. 4G–I). No appreciable differences were observed histologically or when specific markers were examined using qRT-PCR. To assess if MSCs from either mouse strain could contribute to cartilage repair *in vivo*, the MSCs were transduced with a lentiviral GFP (Supplementary Figure 3A,B). After transfection, no silencing of the GFP was observed within the cells, no decreased in differentiation potential was observed (Supplementary Figure 3C–E), and additionally no toxicity was observed when cells were stained with EthD-1 (data not shown).

### Contribution of Synovial MSCs to Cartilage Repair *In Vivo*

**Figure 5:**
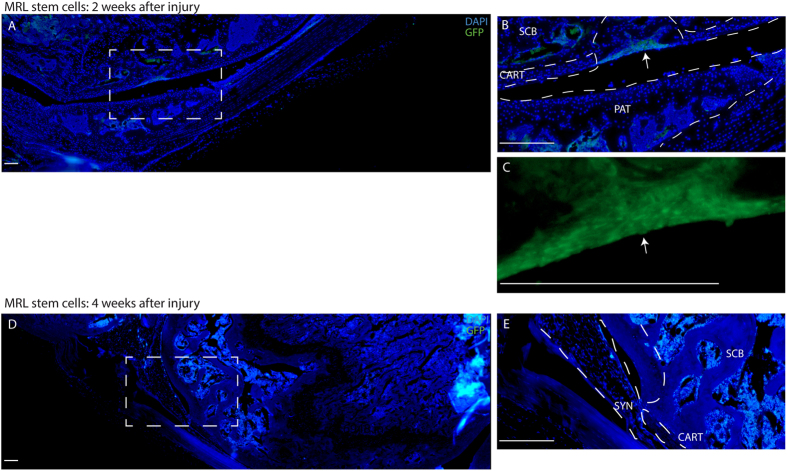
Exogenous delivery of MRL MSCs contribute to Cartilage Repair. MRL synovial GFP^+^ MSCs migrate to the defect within 2 weeks after injury (**A**) and directly contribute to cartilage repair (arrows **B**,**C**). By 4 weeks after injury, no GFP can be detected at the injury site. Scale bars = 200 μm. SCB = sub-chondral bone, CART = cartilage, SYN = synovium, PAT = patella.

In C57BL6 mice injected with MRL synovial GFP^+^MSCs, positive GFP staining was observed in sub-chondral bone, patella and within the cartilage defect at 2 weeks (Fig. 5). Furthermore, it appeared that the injected MSCs were directly contributing to repair of the defect (Fig. 5B,C – higher magnification, DAPI channel removed). By 4 weeks after injury, no GFP positive staining was observed in the joint (Fig. 5D,E). A section of liver from a constitutive GFP mouse was used as a positive control (Supplementary Figure 3F), while a knee joint from a non-GFP mouse was used as the negative control (Supplementary Figure 3G).

**Figure 6:**
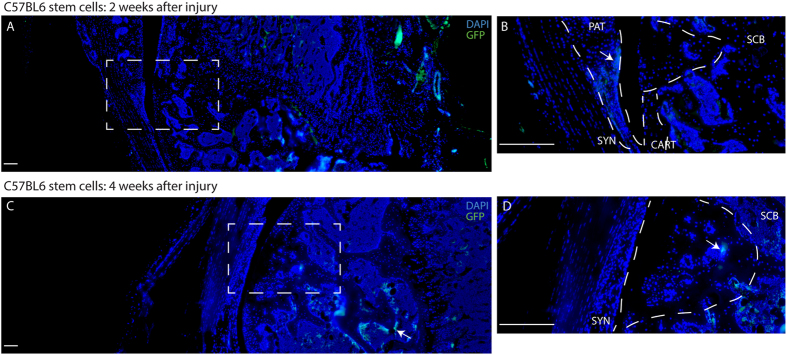
Exogenous delivery of C57BL6 MSCs in a mouse cartilage injury model. C57BL6 synovial GFP^+^ MSCs were not found in the defect within 2 weeks after injury (**A**,**B**), but were observed with the patella and adjacent synovium (arrow **B**). By 4 weeks after injury, GFP positive cells were observed in the defect site (arrow **D**), and below the defect in the sub-chondral bone (arrow **C**). Scale bars = 200 μm. SCB = sub-chondral bone, CART = cartilage, SYN = synovium, PAT = patella.

In contrast to injected MRL MSCs, injected C57BL6 GFP + MSCs were not observed in the cartilage defect at 2 weeks after injury (Fig. 6); however, some GFP positive staining was observed in the patella, synovium and sub-chondral bone (Fig. 6B). At 4 weeks after injury, few C57BL6 MSCs were observed in or surrounding the defect (Fig. 6D), and increased GFP staining was observed within the sub-chondral bone (Fig. 6C,D).

To determine if the repaired tissue demonstrated properties of articular cartilage, collagen 2 staining was performed on the sections. In uninjured joints, collagen 2 staining was observed at the surface of the cartilage as expected (Supplementary Figure 4A,E). In injured but untreated C57BL6 mice (2 or 4 weeks after injury), minimal staining was observed in the defect site (Supplementary Figure 4C,D), in contrast to MRL mice, which demonstrate robust collagen 2 staining in the defect site at both time points (Supplementary Figure 4G,H). In C57BL6 mice that received C57BL6 MSCs after injury, collagen 2 staining was observed in the defect site, (Supplementary Figure 4I,J), and the staining was more pronounced in C57BL6 mice that received MRL MSCs after injury (Supplementary Figure 4K,L).

### Quantification and Non-Invasive Tracking of Cartilage Repair

**Figure 7:**
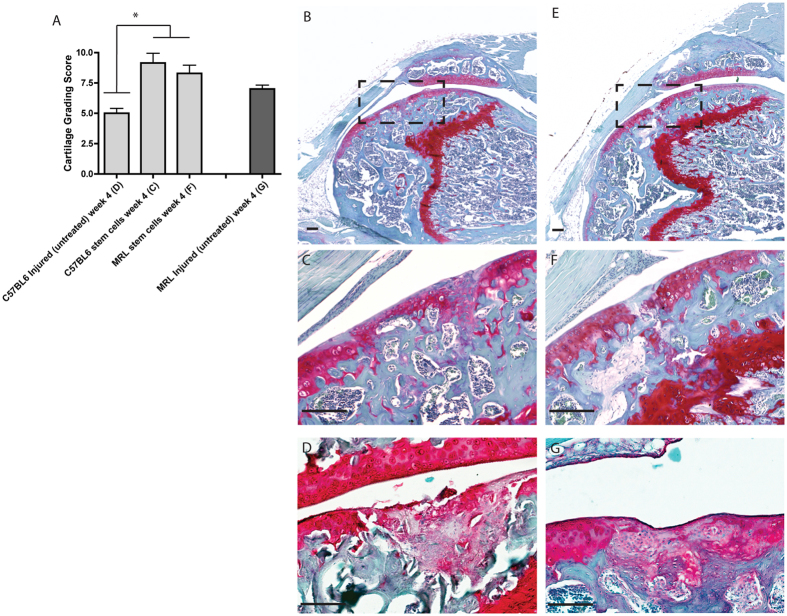
Quantification of Cartilage Injury Repair. Cartilage injuries were graded in C57BL6 mice, MRL mice, and C57BL6 mice that received an injection of either MRL or C57BL6 MSCs (**A**). No difference was observed between MRL and C57BL6 MSC injected groups, but both were significantly better than injured-untreated C57BL6 mice. Representative images are shown of knees injured and injected with C57BL6 (**B**,**C**) or MRL (**D**,**E**) MSCs, or C56BL6 (**D**) and MRL (**G**) knee that have been injured but not injected with MSCs at the 4 week timpoint. Scale bars = 100 μm (**B**,**D**); = 150 μm (**C**,**E**).

To quantify cartilage repair, histological samples from injured (untreated) C57BL6 and MRL mice were compared to C57BL6 mice that had been injured and injected with either C57BL6 or MRL synovial GFP + MSCs (Fig. 7A). While no significant difference in cartilage healing scores was observed at 4 weeks post injury between mice injected with C57BL6 or MRL MSCs, both groups demonstrated increased cartilage repair compared to injured C57BL6 controls that had not received MSCs (Fig. 7A). C57BL6 mice injected with either C57BL6 (Fig. 7B,C) or MRL (Fig. 7D,E) synovial MSCs demonstrated robust cartilage repair, with new proteoglycan positive matrix present in the defect area.

**Figure 8:**
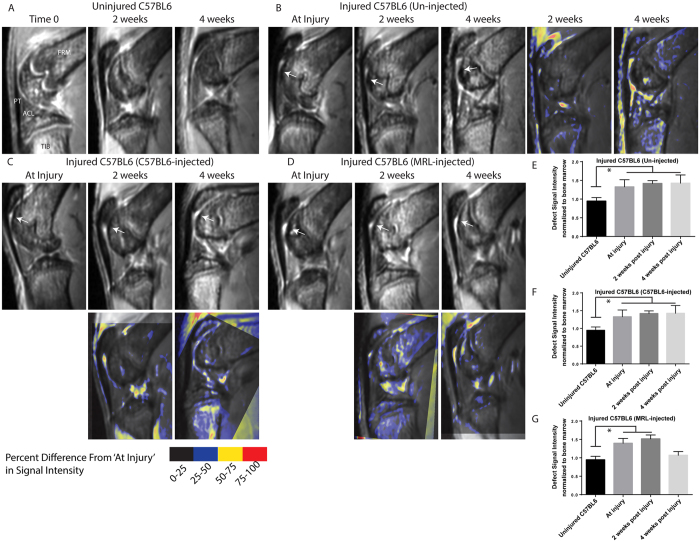
Repeated Measures MRI Tracking of Cartilage Injury Repair Over Time. MRI was used to follow repair *in vivo* over 4 weeks in: control joints without injury (**A**), C57BL6 injured mice without MSC injection (**B**), C57BL6 injected with C57BL6-derived MSCs (**C**) and C57BL6 injected with MRL-derived MSCs (**D**). Arrows indicate the position of the defect. Images at 2 or 4 weeks after injury were registered (using the defect site) to the images collected at the ‘At Injury’ time-point and pseudo-coloured to demonstrate difference in signal intensity between the two time-points. Signal intensity of the defect was quantified at injury, 2 weeks and 4 weeks post injury for mice that were un-injected (**E**), injected with C57BL6 MSCs (**F**), or MRL MSCs (**G**). A significant reduction in signal intensity was only observed 4 weeks after injection with MRL MSCs. FMR = Femur, TIB = Tibia, ACL = Anterior Cruciate Ligament, PT = Patellar Tendon.

To track cartilage repair over time, uninjured C57BL6 (Fig. 8A), injured-untreated C57BL6 (Fig. 8B), injured C57BL6 mice injected with C57BL6 MSCs (Fig. 8C) and injured C57BL6 mice injected with MRL MSCs (Fig. 8D) were imaged at 0, 2 and 4 weeks using magnetic resonance imaging (MRI). The images acquired at 2 or 4 weeks were registered with the defect site in the ‘at injury’ time-point and pseudo-coloured to represent the difference in signal intensity at a given pixel. In the injured-untreated C57BL6 mice, degradative changes in the joint were observed at 2 weeks that progressed by 4 weeks (Fig. 8B), and this can be seen in the pseudo-coloured registered image showing a small yellow area around the defect at 2 weeks that has grown to cover most the femoral cartilage surface by 4 weeks (Fig. 8B). However, joints from C57BL6 mice injected with either C57BL6 or MRL synovial MSCs more closely resemble the control, uninjured joints at 4 weeks post injury, and this can also be observed in the respective pseudo-coloured registered images for C57BL6- (Fig. 8C) or MRL- (Fig. 8D) injected joints. The signal intensity in the defect site was quantified in all groups as previously described^29^ and injured-untreated C57BL6 mice displayed a significant increase in signal intensity after injury that was still present 2 and 4 weeks after injury (Fig. 8E). When C57BL6 MSCs were injected into an injured joint, no difference in signal intensity was observed compared to the untreated group (Fig. 8F). When MRL MSCs were injected into the joint, a significant decrease in signal intensity at weeks was observed and there was no significant difference in signal intensity between uninjured cartilage and MRL MSC injected joint cartilage by 4 weeks post injury (Fig. 8G). The MRL injected C57BL6 mice showed the greatest change in signal intensity in the defect site in comparison to imaging directly after injury. When examined in the context of the histological data (Fig. 7), this suggests that these mice demonstrated the greatest cartilage repair.

## Discussion

In this study, we sought to determine whether MRL/MpJ-derived synovial MSCs demonstrated an enhanced capacity for repairing focal, full-thickness cartilage defects compared to C57BL6-derived synovial MSCs. A number of previous studies involving intra-articular injection of exogenous cells have observed varying levels of cartilage repair coupled with low numbers of engrafted cells at the injury site^25^,^30^,^31^. In the 2012 paper by Diekman *et al.*^25^, the authors hypothesized that injection of MSCs could prevent post-traumatic arthritis (PTA) in mice^25^. The authors also hypothesized that MSCs derived from MRL/MpJ mice would be superior to C57BL6-derived MSCs due to their heightened ability to repair cartilage endogenously in MRL mice. Bone marrow-, but not synovium-derived MSCs were isolated using the markers CD45−/TERR119−/PDGFRα + /Sca-1+. The cells were cultured at 2% oxygen, which the authors suggested enhanced the differentiation potential of the cells. The authors also observed that a single injection of 10,000 cells, irrespective of which mouse strain the cells were isolated from (MRL vs. C57BL6), was able to prevent PTA up to 8 weeks. However, only a small proportion of the cells were observed to engraft.

When we assessed the ability of either MRL- or C57BL6-derived synovial MSCs to repair focal cartilage defects, as with Diekman *et al.*^25^, we observed that there was little difference in outcome between the two treatment groups: defects in both treatment groups were not fully repaired although we did see extensive preservation of proteoglycan-rich extracellular matrix adjacent to the defect. Interestingly, however, at 2 weeks post-injury we observed MRL-derived MSCs at the defect site that appeared to be directly contributing to cartilage repair, while this was not observed in mice injected with C57BL6-derived synovial MSCs. Furthermore, by 4 weeks, no GFP was detected in the defects of either group. This observation might be partly explained by the known different cellular response to injury in MRL and C57BL6 mice. In MRL mice, we observed an increase in MSCs and macrophages at the site of injury immediately after the injury occurred, with a decrease in staining after 2 and 4 weeks, while in C57BL6 mice, fewer cells were observed directly after injury, but staining increased at 2 and 4 weeks. This may suggest that MRL cells (stem and/or immune) are ‘primed’ and in a readied state to respond immediately after injury. After this quick response to injury, the absence of GFP positive cells at later time points suggests that they may differentiate (silencing the GFP), migrate away from the defect area, or die. In C57CL6 mice, however, it might take more time to accumulate MSCs and/or immune cells to the area of injury. Additionally, the ‘muted’ inflammatory response previously observed in MRL^32^,^33^,^34^ mice may be playing a role in regulating the migration, proliferation and differentiation of both MSCs and macrophages^35^,^36^. In this study, it was observed that MRL mice demonstrated a decrease in MSC marker expression (specifically in CD140a) *in vivo* over time after injury, which might signify that the cells are differentiating and losing “stemness” markers. This hypothesis is in alignment with our data demonstrating that MRL-derived MSCs can integrate into the defect within 2 weeks after injury, potentially undergoing differentiation. In regards to the role of immune cells in the enhanced healing ability of MRL mice, and the observation that MSC injection alone can improve cartilage healing in a non-healing background (C57BL6), we have recently demonstrated that bone marrow transfer from MRL to C57BL6 does not lead to an improved healing outcome after cartilage injury^37^. This suggests that while macrophages and other immune cells may play a role in MRL tissue regeneration, they are not sufficient to induce tissue repair in the absence of MSCs.

To summarize, the objective of this study was to determine whether MRL- and C57BL6-derived synovial MSCs were equivalent in promoting focal cartilage defect repair. We did not observe any significantly different effects of the treatment effect between MRL- and C57BL6-derived cells (with the exception of MRI signal intensity). Both MRL and C57BL6 MSC injected groups displayed a significant beneficial effect compared to injured-untreated groups. All MSC injected animals showed better preservation of cartilage proteoglycan levels, even though repair of the defects remained incomplete at 4 weeks post injury. These results, along with previously published studies, suggest that exogenous delivery of MSCs for articular cartilage repair may be a viable treatment option for patients with injury and/or arthritis.

It is interesting that while both MRL and C57BL6 MSCs ultimately enhanced cartilage repair, evidence of contribution to cartilage repair (e.g. homing to the wound) was only observed for MRL-derived stem cells. It should also be made clear that in both strains of mice, none of the repaired cartilage tissues contained any GFP+ cells at 4 weeks after injury. This suggests that different strains of mice may have different cellular mechanisms to deal with wound healing, potentially through a differential regulation of inflammation after injury. Testing this hypothesis would require further study.

As with all experimental studies, this one has assumptions and limitations. First, the sample size used for the studies was small, yet was still able to reveal some statistically significant differences between groups. Second, whereas the stoppered needle limits the depth of needle penetration into the cartilage, it does not allow full control of the angulation of the defect, which can influence defect size and depth and potentially bias cartilage grading scores^29^. Third, the heterogeneity of Sca-1 purified cells is a potential limitation. Since mouse MSC markers are still debated in the literature, some MSC heterogeneity could influence our experiments based on variation in the number of ‘true’ multipotent MSCs in each injection. Lastly, the choice of time-points was arbitrary, but aimed to study early changes in the joint after injury. It is possible that a number of significant effects were missed in the first week, or after the 4^th^ week. Obviously, a larger numbers of animals, more time-points, and longer intervals would improve the resolution of some of the possible interactions between immune and stem cells.

Taken together, the experiments presented in this study show that mouse synovial MSCs purified with Sca-1 have the capacity for chondrogenic differentiation *in vitro* and *in vivo*. Intraarticular injection of these Sca-1+ synovial MSCs from MRL or C57BL6 mice protects against the joint deterioration that would normally result after a surgically induced focal cartilage defect. Although the mechanism of protection does appear to be different between the two strains of mice, this observation requires further study to elucidate the mechanism behind it.

## Materials and Methods

### Ethics Statement

Animal studies were carried out in accordance with the recommendations in the Canadian Council on Animal Care Guidelines. Animal protocols and surgical procedures in this study were approved by the University of Calgary Health Sciences Animal Care Committee.

### Experimental Outline

Focal cartilage defects were surgically induced in the left knee of 4–6 week old mice (n = 52) as detailed below. C57BL6 (n = 43) and MRL (n = 9) mice were allocated into experimental groups for histological analysis of defect healing, immunohistochemistry for MSC markers, or MRI as indicated in Supplementary Figure 5. An additional 2 mice of each strain were used as donor animals to establish synovial MSC cell cultures.

### Focal Cartilage Defect

A hypodermic needle was used to induce a standardized cartilage defect after Eltawil *et al.*^24^ and adapted by our group^29^. A depth gauge ‘stopper’ in the form of a bead was made from Apoxie Sculpt® and placed on a 26 gauge (26G) needle (BD Biosciences) such that 1.5 mm of the tip was exposed. Previously, we have characterized the defect with a width of 367 μm ( + /−123 μm) and depth of 1071 μm ( = /−41 μm)^20^. For sterilization, stopped needles were exposed to UV radiation for 15 minutes. Mice were anaesthetized with isoflurane (2.5 L/min) for the duration of the surgery. The hair around the knee joint was clipped followed by swabbing of the surgical area with betadine. A small skin incision on the medial side of the left knee was made to expose the patella and the patellar tendon. Keeping the knee extended, the tip of stopped needle was inserted under the patellar tendon from the medial side and aimed towards the femur. Pressure was applied with a twisting motion until the tissue was at the hilt of the stopper. The dislodged cartilage was removed, sterile gauze was used to blot excessive bleeding and the incision was closed using wound clips. The surgical area was then swabbed with gentamycin to prevent infection. Animals received buprenorphine (0.05 mg/kg) at the conclusion of the surgery as well as the next day for pain management. At the experimental endpoints, animals were euthanized, both legs were disarticulated at the hip and fixed with 10% neutral buffered formalin (NBF).

### Magnetic Resonance Imaging (MRI)

Cartilage injuries were imaged *in vivo* at 0, 2 and 4 weeks after injury using a 9.4 T/21 cm horizontal bore magnet (Magnex, UK) with a Biospec console (Bruker, Germany) and a cryogenic transceive quadrature RF surface coil (CryoProbe, Bruker, Germany). A spin echo, four-segment Rapid Acquisition with Relaxation Enhancement (RARE) sequence was used with the following parameters: RARE factor = 4, Repetition time = 2000 ms, echo time = 9.346 ms, FOV = 1.92 cm, slice thickness = 0.25 mm, matrix = 256 × 256, averages = 12^29^. Signal intensity was quantified using SPIN (Magnetic Resonance Imaging Institute for Biomedical Research, SVN Revision 2131). The signal intensity of the defect was acquired and then normalized to the signal intensity of the bone marrow in the same mouse. The signal intensity of the defect area was then normalized to the bone marrow signal intensities obtained from each strain of mouse at each time point. Images from repeated measures on the same animal were co-registered at the defect site and pseudo-coloured in Adobe Photoshop based on absolute differences in signal intensity from the time 0 control. After the final imaging at 4 weeks, mice were euthanized, and knee joints were harvested for histological analysis.

### Histological Analysis

Hind-limbs were dis-articulated at the hip and fixed for 3 days in 10% NBF, then decalcified in 10% EDTA for three weeks. After decalcification was complete, samples were washed with water and underwent tissue processing and embedding. Paraffin-embedded sagittal sections at 10 μm thick were stained with Safranin-O/fast green and graded for cartilage defect healing using a scoring matrix previously described^22^. Briefly, cartilage repair in all mouse groups was evaluated after 4 weeks by scoring the full-thickness defect for cell morphology (0–4), matrix staining (0–3), surface regularity (0–3), thickness of cartilage (0–2) and integration with native cartilage (0–2). On this scale, a newly created FTCD has an overall score of 0 and the (maximal) overall score for uninjured native cartilage is 14. The cell morphology score (0–4) is an indicator of the type of tissue that has filled the defect: a score of 4 indicates complete regeneration of smooth-surfaced hyaline cartilage, while lower scores are given for defects filled with fibrocartilaginous or non-cartilage tissue. The matrix staining score (0–3) measures the amount of proteoglycan present in the cartilage defect: a score of 3 indicates dense continuous staining throughout the thickness of the cartilage as in uninjured joints.

### Immunohistochemistry

Sagittal sections (10 μm) were cut, deparaffinized in CitriSolv (Fisher Scientific; Fairlawn, NJ) and rehydrated through a series of graded ethanol to distilled water steps. Antigen retrieval steps (10 mM sodium citrate, pH 6.0) and blocking (1:500 dilution; 100 μL rat serum:50 mL TRIS-buffered saline, 0.1% Tween 20 (TBST) for 1 hr) were performed prior to going through sequential wash (TBST) and primary antibody application steps. Primary antibodies for Sca-1 FITC (BD Biosciences), F4/80 PE (BD Biosciences) and CD140a PE (Biosciences) and the nucleic acid stain DAPI (Sigma) were applied to sections. After antibody staining, sections were mounted using FluorSave reagent (Calbiochem) and coverslipped.

### Imaging

Slides were imaged using a Plan-Apochromat objective (20×/0.8 M27) on an Axio Scan.Z1 Slide Scanner microscope (Carl Zeiss); DAPI (353 nm/465 nm), FITC (493 nm/517 nm), and phycoerythrin (PE) (565 nm/576 nm).

### Isolating Murine Synovial Cells

At necropsy, the joint space was exposed under a dissecting microscope. Synovial tissue was dissected out from the joint and placed in a petri dish containing PBS and 1% anti-anti (Life Technologies). Grooves/scratches were made in the bottom of a 12 well plate with a scalpel to improve tissue transfer efficiency and to promote tissue adherence. Tissue samples were transferred to the 12 well plate and MSC expansion medium, consisting of DMEM/F-12 (Biowhittaker), 10% fetal bovine serum (Life Technologies), 1% non-essential amino acid (Life Technologies), 1% anti-anti (Life Technologies), and 0.2% beta-mercaptoethanol (Life Technologies) was added to each well. Cells were then incubated at 37 °C in 2% O_2_ and 5% CO_2_. Medium was changed every 2–3 days until outgrowth was observed from the tissue, after which medium changes were performed daily. When cells that had outgrown from the tissue had reached confluency, the tissue piece was removed and the cells were passaged into a T25 flask. Subsequent medium changes occurred every 2–3 days, or as required.

### Sca-1 Selection of Cells

Medium was removed and cells were trypsinized with 0.25% trypsin-EDTA (Life Technologies) until cells were seen to lift from the plate, after which medium was added to deactivate the trypsin. The cell suspension was then centrifuged for 5 minutes. A working solution of BD IMAG buffer (BD Biosciences) was produced by mixing 1 mL of BD IMAG Buffer (10×) with 9 mL of sterile distilled water. The solution was kept on ice for the duration of the procedure. After centrifugation, the supernatant was discarded and the cell pellet was resuspended in 1 ml of diluted BD buffer. Five micro-litres of BD immune cell depletion cocktail was added to the cell suspension, placed on ice for 15 minutes after which 5 μL of magnetic particles (BD) was added. The suspension was placed in a sorting magnet (BD) for 7 minutes after which the liquid phase was removed and the process was repeated using a Sca-1 positive magnetic selection approach. The method was the same as above but using a Sca-1 biotinylated antibody (EBioscience).

### Tri-lineage (Adipo-, Osteo-, and Chondrogenic) Differentiation and Analysis

For all three differentiation methods, a total of 10,000 cells/well/24well plate were used. For chondrogenic and osteogenic differentiation, cells were seeded into the wells of a 24 well plate using the hanging drop method. The next day, 1 ml of chondrogenic or osteogenic medium was added to each well. For adipogenic differentiation, 10,000 cells were plated per well in a 24 well plate. Cells were cultured with either MSC expansion medium or differentiation medium at 37 °C with 2% oxygen and medium changes performed every 2–3 days (as needed) for 21 days. Adipogenic Differentiation: Differentiation medium consisted of MSC culture medium with 0.5 mM isobutylmethylxanthine, 1 μM dexamethasone, 10 μM insulin, 200 μM indomethacin (all Sigma). Osteogenic Differentiation: Differentiation medium consisted of MSC culture medium with 0.1 μM dexamethasone and 50 μM ascorbate-2-phosphate. Chondrogenic Differentiation: Differentiation medium consisted of sMSC culture medium with 500 ng/mL BMP-2 (Peprotech, Rocky Hill, NJ), 10 ng/mL TGF-β3 (Peprotech), 10-8 M dexamethasone (Sigma, St. Louis, MO), 50 μg/mL ascorbic acid (Sigma), 40 μg/mL proline (Invitrogen), 100  μg/mL pyruvate (Sigma) and supplemented with insulin, transferrin, and selenium (Sigma).

Resultant cells were fixed in 4% paraformaldehyde overnight, then stained with Alcian blue, Alizarin red and Oil red O (all Sigma) to detect glycosaminoglycans (cartilage), calcium (bone) and lipids (fat) respectively.

Gene expression within MSCs was evaluated in each of the treatment groups by quantifying the relative gene expression of chondrogenic markers Sox9, Col2A1, and ACAN; adipogenic markers PPAR-gamma, ADIPOQ and aP2; and osteogenic markers SP7, SPARC, Runx2 using quantitative polymerase chain reaction (qPCR). Total mRNA was extracted and purified using Trizol in accordance with the manufacturer’s instructions (Invitrogen, Carlsbad, CA) and converted into cDNA according to the High Capacity cDNA Reverse Transcription Kit protocol (Applied Biosystems, Foster City, CA, USA). Fifty nanograms of cDNA was added to a 96-well qPCR plate along with TaqMan® validated probes/primers and TaqMan® Universal PCR Master Mix. In addition, an 18S RNA probe/primer was used as an internal control. Two biological replicates (cell culture) and methodological triplicates (PCR reaction) of each time point (undifferentiated/control and after differentiation) were performed, and all cDNA used was from the same corresponding replicate to reduce variability. To calculate fold change, the ddCT method was used and all values were normalized against undifferentiated MSCs.

### GFP Lentiviral Transduction

At passage 2, Sca-1 purified synovial cells were incubated with the following mix overnight (~12 h) at 37 °C, 2% O_2_: 5 mL MSC expansion medium, 5 μL GFP lentivirus (where GFP is driven by the EF1-alpha promoter), and 2 μL of Polybrene (8 μg/mL, Sigma). Medium was changed the following day.

### Intra articular Injection of Synovial MSCs

Intraarticular injections of Sca-1 + GFP+ synovial cells from either C57BL6 or MRL/MpJ mice, or sterile PBS (as a vehicle control) were performed into the knees of male C57BL6 mice aged 4–6 weeks. Mice were prepared for surgery as described above. With the mouse in a supine position, the knee was placed into a custom made holder such that the fixation screws were aligned just above the patella tendon. The screws were tightened superior to the lateral and medial epicondyles to immobilize the femur. A small skin incision was made to expose the patella and the tendon. Using the micromanipulator, a 30G needle was inserted through the tendon and into the space between the patella and the femur. Entry into the joint space was considered successful when the patella could be seen lifting upwards as the needle slid underneath. The total injection was 2 μL containing 10,000 cells. For animals undergoing MRI scans, incisions were closed using 5-0 Ethilon nylon suture. For all other animals, incisions were closed using wound clips.

### Statistical Analysis

Standard descriptive statistics were used to summarize the variables, i.e. means and standard deviation. p ≤ 0.05 was considered to be significant. Statistical analysis was performed by ANOVA followed by Bonferroni’s post hoc testing. Statistical methods were reviewed by a biostatistician at the University of Calgary.

## Additional Information

**How to cite this article**: Mak, J. *et al.* Intra-articular injection of synovial mesenchymal stem cells improves cartilage repair in a mouse injury model. *Sci. Rep.* **6**, 23076; doi: 10.1038/srep23076 (2016).

## Supplementary Material

Supplementary Information
