# Supplementary material for: Intra-articular injection of synovial mesenchymal stem cells improves cartilage repair in a mouse injury model
Source: Sci Rep. 2016 Mar 17;6:23076. doi: 10.1038/srep23076 (PMC4794799; doi:10.1038/srep23076)
Supplement: Supplementary Information [file srep23076-s1.pdf]

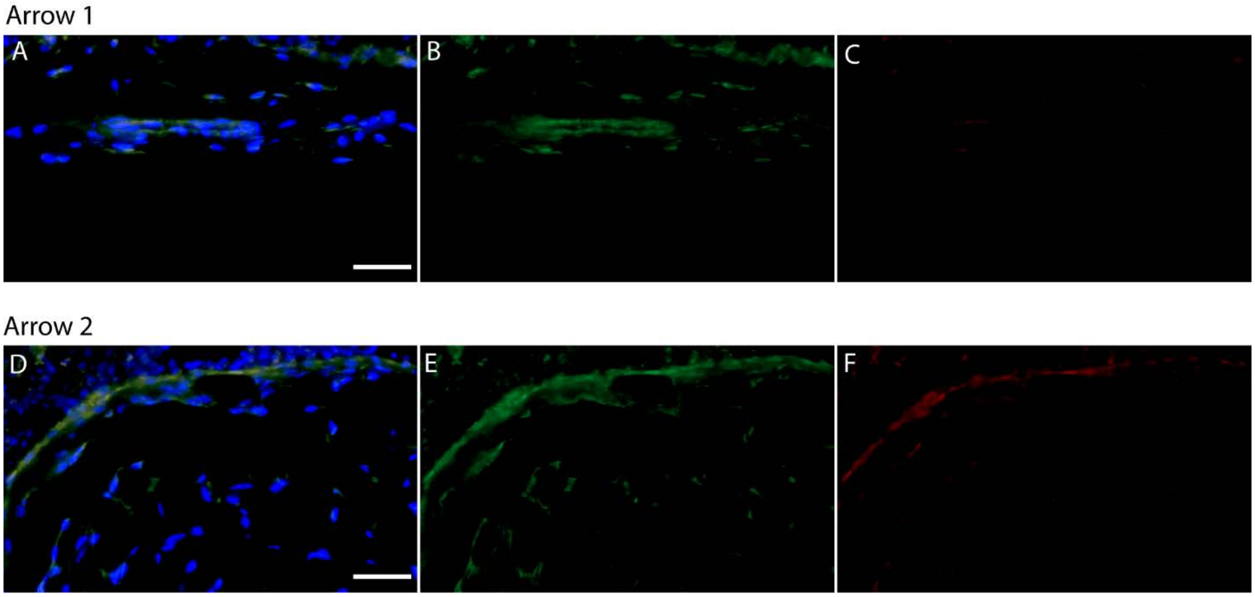

Supplementary Figure 1. High Resolution images of cells described in Figure 1. Sca-1+CD140a- cells can be observed in the synovium near the defect area (A merge, B Sca-1, C CD140a), while Sca-1+CD140+ double positive cells are observed within the synovium directly adjacent to the defect (D merge, E Sca-1, F CD140a). DAPI = blue, Sca-1 = green, CD140a = red. Scale bars = 20µm.

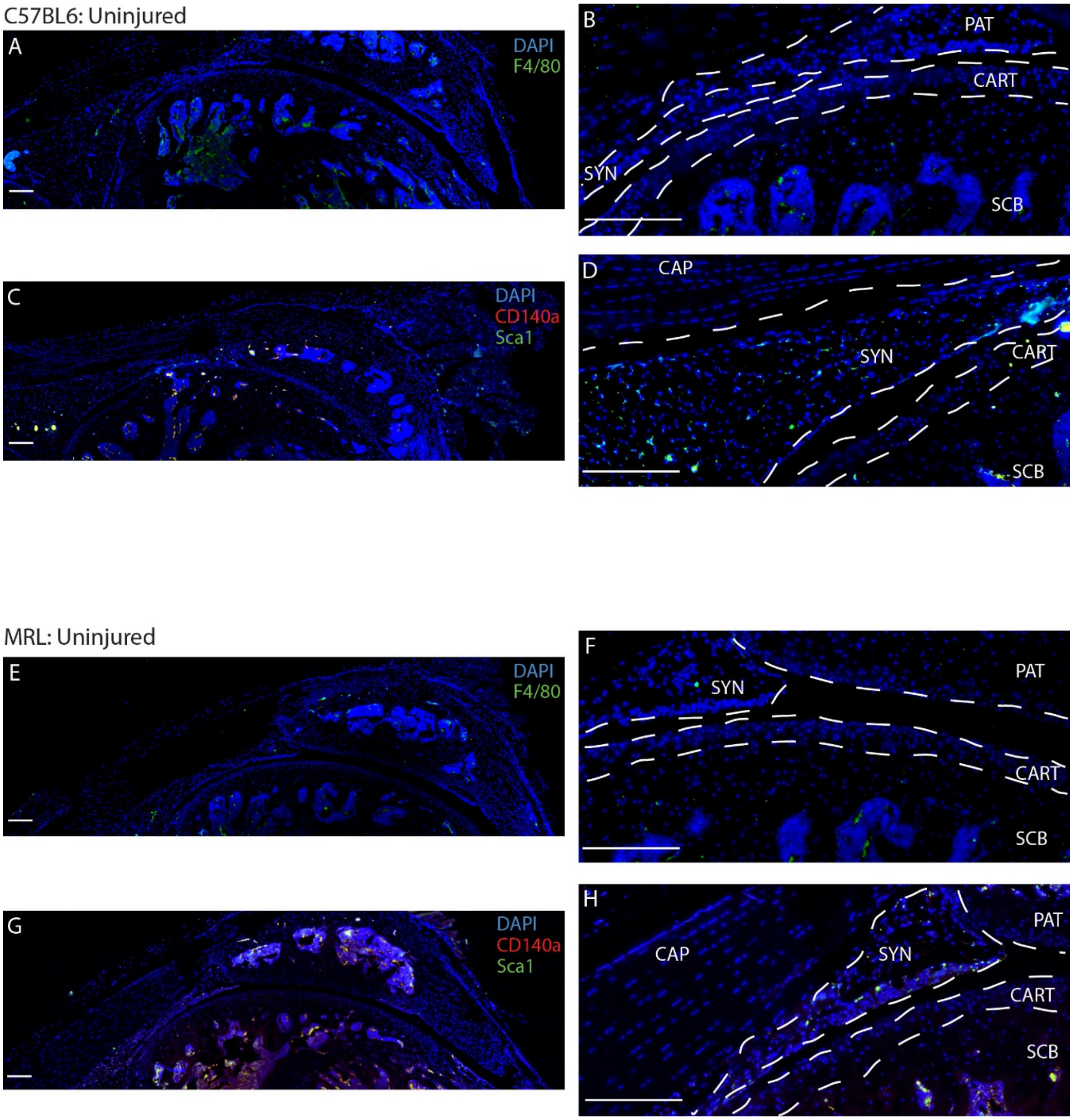

Supplementary Figure 2. MSC and macrophage identification in uninjured joints. In uninjured C57BL6 mice, F4/80 positive cells can be observed in the sub-chondronal bone, while cells positive for CD140a and Sca-1 can also be observed in the sub-chondral bone, but only Sca-1 positive cells are observed in the synovium. In uninjured MRL mice, few F4/80 positive cells can be observed in the sub-chondronal bone, while cells positive for CD140a and Sca-1 can also be observed in the sub-chondral bone, but again, only Sca-1 positive cells are observed in the synovium. Scale bars = 200µm. SCB = sub-chondral bone, CART = cartilage, SYN = synovium, PAT = patella, CAP = capsule.

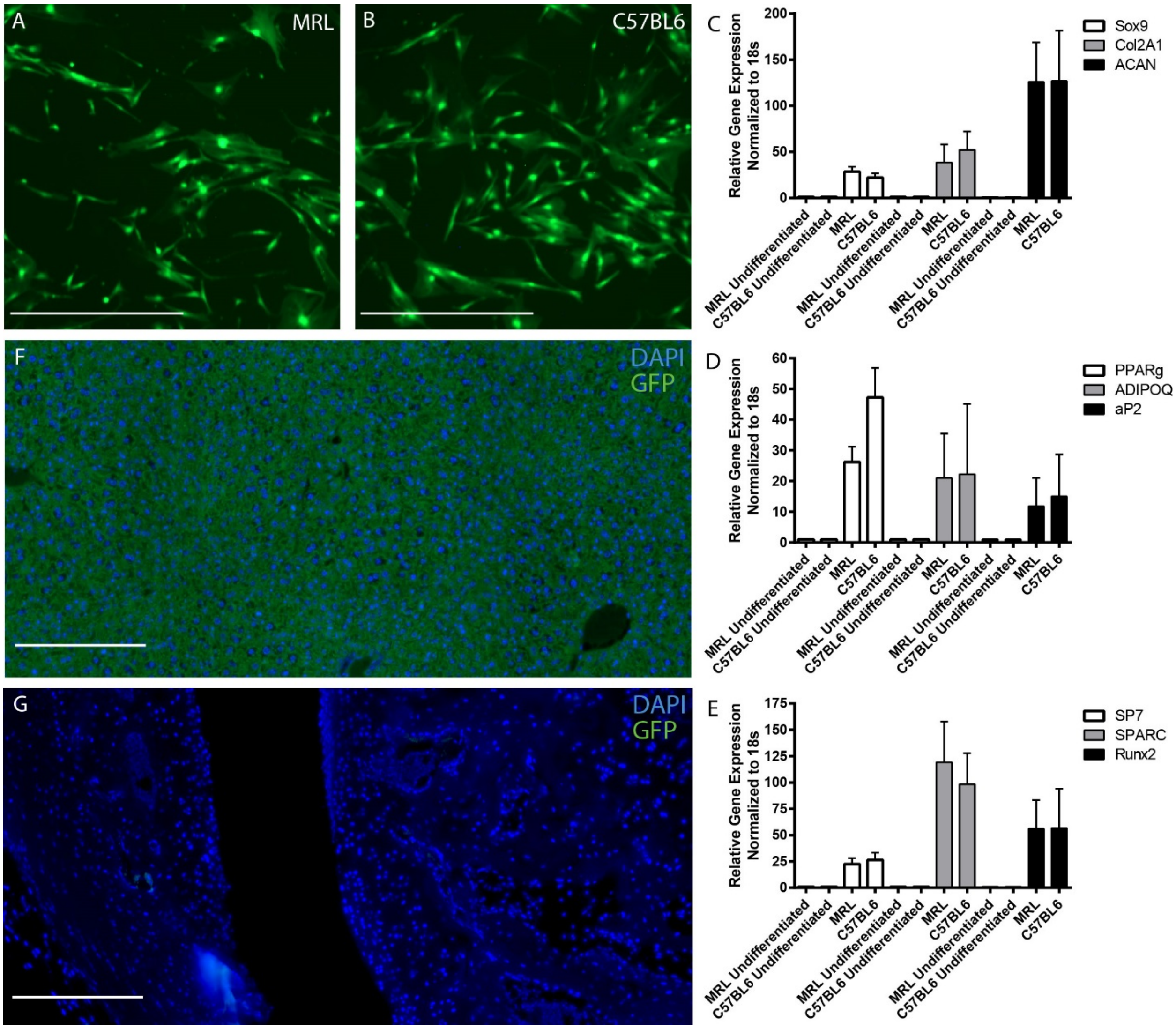

Supplementary Figure 3. Positive and negative controls for GFP. MSCs from MRL (A) or C57BL6 (B) mice were also transduced with a lentiviral GFP marker. No loss in differentiation potential into chondrocytes (C), adipocytes (D) or osteoblasts (E) was observed after GFP transduction. A liver sample from a constitutively expressing GFP mouse (F) and knee joint from a non-GFP/non-GFP injected mouse (G) were used to control for auto-fluorescence when imaging for GFP staining. Scale bars = 100µm for A,B; 200µm for C,D.

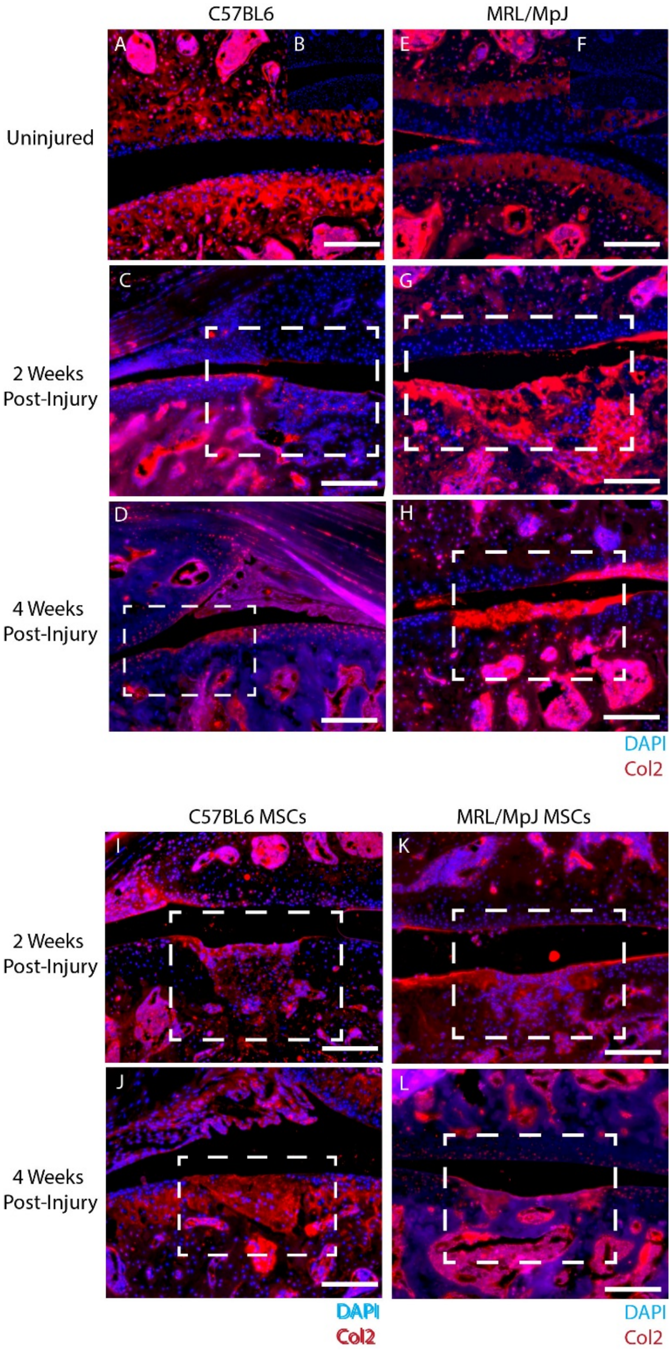

Supplementary Figure 4. Collagen 2 staining of articular cartilage injuries. Un-injured articular cartilage in C57BL6 (A) and MRL (E) mice demonstrates robust collagen 2 staining (secondary controls inset B, F). Two and four weeks after injury, C57BL6 mice (C,D) demonstrate minimal collagen 2 staining, while MRL mice (G,H) demonstrate pronounced staining in the defect area. When injured C57BL6 joints were injected with C57BL6 (I,J) or MRL (K,L) MSCs, collagen 2 staining can be observed in the defect area at all time points examined. Scale bars = 200µm.

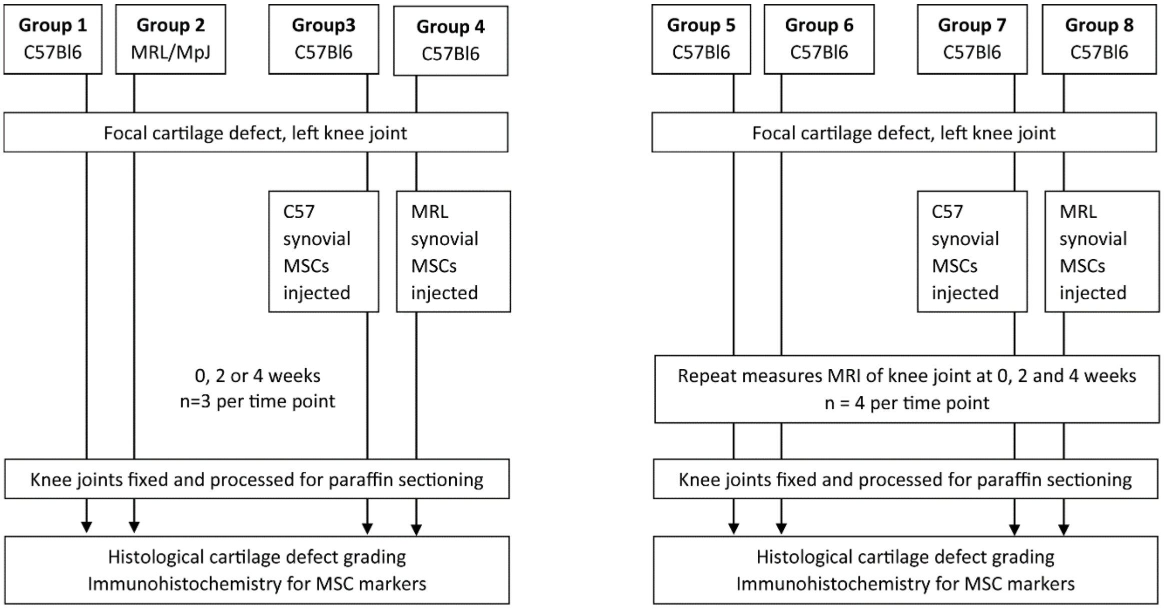

Supplementary Figure 5. Flow chart of experimental design.
